# Patterns of Sediment Fungal Community Dependent on Farming Practices in Aquaculture Ponds

**DOI:** 10.3389/fmicb.2021.542064

**Published:** 2021-02-19

**Authors:** Zhimin Zhang, Qinghui Deng, Xiuyun Cao, Yiyong Zhou, Chunlei Song

**Affiliations:** ^1^Key Laboratory of Algal Biology, State Key Laboratory of Freshwater Ecology and Biotechnology, Institute of Hydrobiology, Chinese Academy of Sciences, Wuhan, China; ^2^University of Chinese Academy of Sciences, Beijing, China

**Keywords:** farm ponds, sediments, fungal community, aquatic ecosystems, environmental factors

## Abstract

Despite fungi playing an important role in nutrient decomposition in aquatic ecosystems and being considered as vital actors in the ecological processes, they received limited attention regarding the community in aquaculture pond sediments which are extremely important and typically disturbed habitats. Using an ITS1 region of fungal rDNA, this study aimed to investigate sediment fungal communities in fish, crab, and crayfish ponds for decades of farming practices at representative aquaculture regions in the middle Yangtze River basin, China. We then aimed to explore the community patterns associated with species-based farming practices in the ponds at 18 farms. The results showed that the pond sediments harbored more than 9,000 operational taxonomic units. The sediments had significantly higher alpha diversity in crab ponds compared to that in fish and crayfish ponds. The fungal phyla largely belonged to *Ascomycota* and *Chytridiomycota*, and the dominance of *Rozellomycota* over *Basidiomycota* and *Aphelidiomycota* was observed. The majority of sediment fungal members were ascribed to unclassified fungi, with higher proportions in fish ponds than crab and crayfish ponds. Further, the fungal communities were markedly distinct among the three types of ponds, suggesting divergent patterns of fungal community assemblages caused by farming practices in aquaculture ponds. The community diversity and structure were closely correlated to sediment properties, especially sediment carbon content and pH. Thus, the distribution and pattern of fungal communities in the sediments appear to primarily depend on species-based farming practices responsible for the resulting sediment carbon content and pH in aquaculture ponds. This study provides a detailed snapshot and extension of understanding fungal community structure and variability in pond ecosystems, highlighting the impacts of farming practices on the assembly and succession of sediment fungal communities in aquaculture ponds.

## Introduction

Fungi are important members of the ecosystem, covering a wide diversity of lineages that have thrived in biospheres (Richards et al., 2012; Tedersoo et al., 2014) where they comprise non-ignorable proportions of microbial community populations. In recent decades, the development of molecular technology-independent cultivation methods has enabled the characterization of microorganisms including bacteria and fungi in many ecosystems, providing unprecedented insight into microbial diversity, spatial distributions, and functional roles in ecosystems as well as the responses to natural disturbances and anthropogenic activities (Wu et al., 2013; Zhang et al., 2016; Cunliffe et al., 2017; Brodie et al., 2018). However, the previous studies on microorganisms mainly focus on bacteria compared to fungi, involving the ecology in terrestrial environments such as soils. Conversely, in aquatic ecosystems, sediments from different environments possess various properties (Yu et al., 2016) and develop specific ecological niches to make fungi grow in and reside in such habitats, as revealed by the diversification of microbial communities (Tedersoo et al., 2014; Grossart et al., 2019). The fungal community especially in aquatic ecosystems to date is poorly understood, and therefore a theoretical framework to the fungal assemblage pattern and ecology remains to be deeply explored (Grossart et al., 2019).

Freshwater ecosystems are generally subdivided into running (such as lentic lakes and ponds) and standing (such as lotic streams and rivers) waters, with significant differences in fungal communities between lotic and lentic freshwater habitats such as rivers and lakes (Grossart et al., 2019) depending on human activities and climate changes (Wang et al., 2020). This might be attributed to the heterogeneity of sediment substrates and the surrounding environments. In previous studies, a high abundance of sediment fungi has been recognized in aquatic ecosystems (Holguin et al., 2001; Richards et al., 2012; Wu et al., 2013; Luis et al., 2019), benefiting carbon and nutrient cycling, and energy flow (Holguin et al., 2001). Noticeably, aquaculture ponds as lentic freshwater habitats are the heavily affected ecosystems due to aquaculture activities. It has been reported that organic carbon and algal polysaccharides can be utilized by saprotrophic mycoplankton in aquatic ecosystems, showing the effects of aquatic fungi on the flow of organic matter (Bochdansky et al., 2017; Cunliffe et al., 2017). Marine farming practices and fertilization-related agricultural practices can regulate fungal communities in environmental systems (Guo et al., 2015), further suggesting that the fungal community patterns may change with different practices in freshwater ponds.

While farm ponds represent small proportions of aquatic ecosystems, they comprise up to 30% of standing freshwater habitats by area (Downing et al., 2006). Many cultured species (such as carps, tilapias, crabs, and other decapod crustaceans) have different feeding habits and biological characteristics so that species-based farming practices have been widely generalized by the development of species-specific aquafeeds and culture models in aquaculture. In aquaculture, cultured fish assimilate small proportions of nutrients from feeds, suggesting that the majority of nutrients derived from feed residuals and excretions of farmed animals sink into the bottoms of ponds, with biologically complex decomposition and mineralization processes (Herath and Satoh, 2015). Farmed animals have significant differences in their utilization of feed nutrients such as nitrogen and phosphorus, which may be associated with fungal function. For example, studies on fungi by Shoun et al. (1992) and Gao et al. (2020) reported that some taxa had denitrifying activity. Factors shaping the fungal diversity pattern and community structure could be quite different under various environments (Guo et al., 2015; Tian et al., 2018; Yang et al., 2020). Whether the superiority of sediment nutrients shapes the fungal community deserves further studies across different farming practices in freshwater aquaculture ponds.

To better understand the sediment fungal community ecology in ponds, we studied three types of ponds with representative cultured species (fish, crab, and crayfish) having significant differences in feeding habits relevant to feed use and efficiency, and the farming managements such as fertilization, culture period, water depth and other factors in aquaculture. Fish ponds generally have higher water levels and yields than crab and crayfish ponds, whereas the two shellfish ponds have more complex food sources and they are usually planted with aquatic plants. In terms of the complexity and difference in farming practices, we suspected that long-term farming practices based on different cultured animals in the ponds could initiate predictable patterns in fungal communities in the sediments, as potentially suggested by the study on microorganisms (Yang et al., 2020). In this study, we aimed to investigate sediment fungal communities of fish, crab, and crayfish ponds and to further explore the community patterns associated with species-based long-term farming practices. The following questions were proposed and addressed: (1) What were the fungal diversity and community composition in the sediments collected from different aquaculture ponds? (2) Whether the diversity patterns in the fungal communities were associated with species-based farming practices? (3) What were the determining factors of driving fungal succession in the sediments?

## Materials and Methods

### Study Site and Sampling

**Figure 1:**
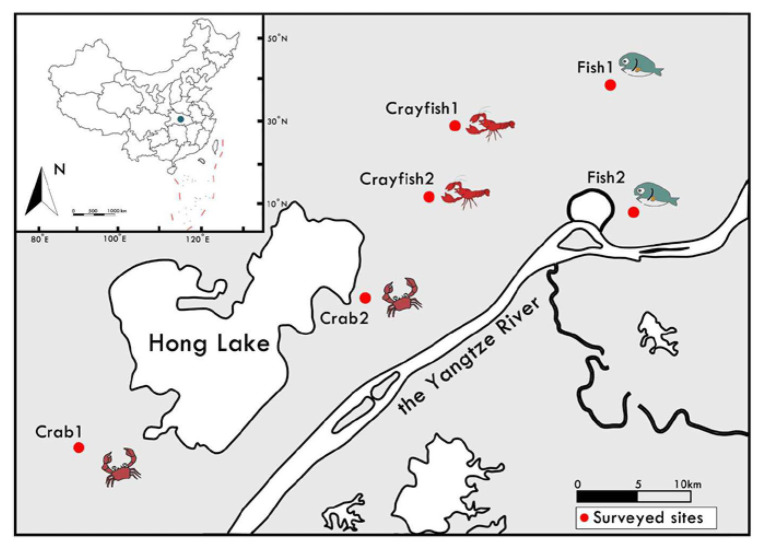
Schematic location and sampling strategy of study sites for sediments from aquaculture ponds around Hong Lake in the middle of Yangtze River Basin, China. For each location, three sediment samples were collected from three aquaculture ponds in three adjacent farms. Each red circle represents a study site where three sediment samples were randomly collected from the same type of three ponds.

In this study, the studied ponds were located around Hong Lake, in the middle Yangtze River basin, China (Figure 1). For the past several decades, this region has been representative of China’s aquaculture and it has various aquaculture ponds. In this study, we collected 18 sediment samples from six locations in the region in January and February 2019 (Figure 1). The sediments were sampled from three types of ponds (fish, crab, and crayfish). All the ponds have been in use for 15–25 years. Since the beginning, the fish and crab ponds were continuously used for fish and crab culture, respectively; the crayfish ponds were used for crayfish culture in the last 10 years. For each location, the same type of three sediment samples were collected from three aquaculture ponds in three adjacent farms using a Peterson grab sampler (about top 10 cm sediment). The same type of farming ponds were very close and grouped geographically. Finally, six sediment samples for each type of pond were obtained in this study. All ponds studied lay fallow during the period so that it could be expected to reflect actual patterns of sediment fungal communities due to the effects of long-term farming practices and avoid sudden disturbances (such as fertilization and prophylactic drug use) during the culture period. After the sediments were thoroughly homogenized, each sediment sample was in duplicate: one subsample for the analysis of sediment properties was kept at 4°C; another subsample for the analysis of fungal communities was stored in liquid nitrogen and then transplanted to −80°C.

### Measurements of Sediment Parameters

Sediment pH was measured by using a pH meter with a glass electrode (Testo 205, Testo, Germany). The sediment samples were dried at 105°C until a constant weight for analysis of the moisture content. Total carbon (TC) in the sediments was determined with a C:N auto-analyzer (multi N/C 3100, analytikjena, Germany). Total nitrogen (TN) was determined with Kjeldahl digestion and distillation azotometry after extraction with concentrated sulfuric acid using an automated Kjeldahl analyzer (VELP Scientifica, Usmate Velate, Italy). Total phosphorus (TP) was digested by HNO_3_, and the concentrations of the digestive solutions were determined with the molybdenum blue method using a UV spectrophotometer (Model 752, Shanghai Modern Science Co. Ltd).

### DNA Extraction and Illumina Sequencing

DNA was extracted from 0.3 g of sediments for each sediment sample using PowerSoil DNA Kit according to the manufacturer’s recommendation (Qiagen, Hilden, NRW, Germany) and then the DNA yield was quantified. In this study, the PCRs were performed by amplifying the ITS region of the ribosomal RNA as follows: 95°C for 5 min, followed by 20 cycles at 95°C for 30 s, 50°C for 30 s and 72°C for 40 s, and a final extension at 72°C for 7 min using primers ITS1F (5'-CTTGGTCATTTAGAGGAAGTAA-3') and ITS2R (5'-GCTGCGTTCTTCATCGATGC-3') with barcodes of an eight-base specific sequence in 10 μl reaction volumes containing 50 ng of DNA template, 5 μl of KOD buffer, 0.2 mM dNTPs (2 μl), 10 μM of each primer (0.3 μl) and 0.2 μl of KOD polymerase. The second round of PCR amplification with the cleaning products above for index and adapters attachment was performed in 20 μl reaction volumes at thermal cycling conditions as follows: 98°C for 30 s, followed by 10 cycles at 98°C for 10 s, 65°C for 30 s and 72°C for 30 s, and a final extension at 72°C for 5 min. The resulting products were excised from 1.8% agarose gel and purified using MinElute PCR Purification Kit (Qiagen, Hilden, NRW, Germany). Subsequently, sequencing libraries were generated, and the libraries were sequenced on Illumina HiSeq2500 platform with a 250 bp paired-end sequencing strategy. Sequence datasets of the 16S rRNA gene in this study are deposited in the NCBI Sequence Read Archive with the accession number PRJNA611895.

### Sequence Processing

Sequences from Illumina HiSeq platform were processed and quality-filtered in the Quantitative Insights Into Microbial Ecology (QIIME) software according to the quality-controlled process (Caporaso et al., 2010). Fungal ITS paired-end reads were merged with a maximum of 20% mismatches in the overlap region using FLASH (Magoč and Salzberg, 2011). The low-quality sequences were removed, and the sequence reads were assigned to the samples based on unique barcodes. High-quality sequences were clustered into operational taxonomic units (OTUs) with 97% similarity cutoff using USEARCH. All chimeras were filtered for discarding singletons, and the low abundance OTUs (at least 0.005% of all sequences) were removed from the datasets (Bokulich et al., 2013). Taxonomic classifications for the fungal ITS sequences were performed with both RDP Classifier against the UNITE database. The most abundant representative sequence of each OTU was subjected to BLASTn searches against the NCBI database with >90% sequence similarity to a reference sequence assigned to the kingdom Fungi. Given the high sequence numbers and the small differences in sequence numbers for all sediment samples in this study, we did not rarefy the samples to equal sequence numbers for downstream analysis. Fungal α-diversity was estimated in the MOTHUR program by the calculated richness (OTUs number) and the phylogenetic diversity of the whole tree (PD whole tree) incorporating the phylogenetic breadth across the taxonomic levels, meanwhile the overall differences in fungal communities among the pond sediment samples were determined by Jaccard and Bray-Curtis distances. The FUNGuild is currently the largest database and it is commonly used to taxonomically assign fungal OTUs into ecological functions for trophic modes of fungi (Nguyen et al., 2016). In this study, only probable and highly probable confidence score guild assignments were used for further analysis.

### Statistical Analyses

In this study, Pearson’s correlation analysis was used to assess the relationships between sediment fungal community diversity and sediment property. The analysis was performed with a value of *p* < 0.05 considered statistically significant using SPSS 20.0. The differences in fungal communities among different sediment samples were calculated using nonparametric permutation-based multivariate analysis of variance (PERMANOVA) and analysis of similarity (ANOSIM) and further were visualized using non-metric multidimensional scaling (NMDS) plots in R programs. The effects of sediment properties on the fungal community structure were tested by the correlation analysis between the fungal community distance matrices and environmental sediment properties using a Mantel test.

## Results

### Pond and Sediment Characteristics

The areas of the selected 18 ponds for sediment sample collections ranged from 3,335 to 21,344 m^2^ with an average of 12,560 ± 8,300, 14,207 ± 2,373, and 5,781 ± 544 m^2^ for fish, crab, and crayfish ponds, respectively. Their averaged water depths were 2.6 ± 0.2, 0.9 ± 0.2, and 0.6 ± 0.1 m. Sediment pH and moisture content in the ponds ranged from 7.07 to 7.75 and from 43.77 to 63.57%, respectively (Table 1). The sediment pH and moisture content were the lowest in fish ponds (pH, 7.19 ± 0.11; Moisture, 52.56 ± 5.45%), followed by crayfish ponds (7.38 ± 0.06; 57.94 ± 2.63%) and crab ponds (7.57 ± 0.10; 59.87 ± 2.61%). Regarding sediment nutrients, TC concentrations were significantly different among the ponds (16.1–30.24 g kg^−1^) with the highest average value in crab ponds (27.46 ± 2.08 g kg^−1^) and the lowest in crayfish ponds (19.17 ± 3.00 g kg^−1^). Similarly, sediments had higher TN and TP concentrations for crab ponds (2.98 ± 0.20 and 1.62 ± 0.36 g kg^−1^), yet the differences were not found between fish ponds and crayfish ponds (Table 1). Sediment C:N values ranged from 7.06 to 10.03. The lowest value was found in crab ponds (8.03 ± 0.71), followed by fish ponds and crayfish ponds (8.79 ± 0.9 and 9.24 ± 0.7).

**Table 1:** Physical and chemical properties, fungal sequencing data, and the fungal diversity for the 18 aquaculture pond sediments examined for this study.

Sediment samples	pH	Moisture (%)	TC (g kg^−1^)	TN (g kg^−1^)	TP (g kg^−1^)	Fungal sequences	OTUs	Coverage (%)	PD
Fish11	7.07	56.92	22.71	2.84	1.04	73,608	1,227	99.80	185.94
Fish12	7.11	48.78	22.02	2.21	0.88	74,085	1,461	99.72	204.90
Fish13	7.20	43.77	18.05	2.10	0.82	73,834	1,081	99.81	186.04
Fish21	7.18	58.12	22.21	2.66	0.99	73,499	1,172	99.80	188.75
Fish22	7.39	55.18	21.36	2.68	1.75	71,570	1,084	99.84	230.16
Fish23	7.17	52.62	21.75	2.21	1.34	73,653	892	99.80	165.16
Crab11	7.58	60.32	29.65	3.06	1.76	69,837	1,509	99.71	450.09
Crab12	7.75	59.76	25.38	2.85	2.19	71,152	1,342	99.75	289.88
Crab13	7.59	58.89	27.58	3.07	1.41	70,817	1,525	99.75	309.27
Crab21	7.49	63.57	30.24	3.11	1.81	66,279	1,449	99.67	241.08
Crab22	7.48	55.65	26.28	2.62	1.24	70,280	1,358	99.72	274.45
Crab23	7.50	61.04	25.61	3.15	1.31	72,773	1,663	99.69	291.41
Crayfish11	7.44	56.74	18.57	2.63	1.15	72,263	1,149	99.77	252.43
Crayfish12	7.32	54.12	16.10	2.01	0.94	70,320	1,084	99.74	199.92
Crayfish13	7.28	61.52	19.42	2.53	1.27	67,527	1,211	99.76	250.86
Crayfish21	7.41	60.01	17.59	2.08	1.12	72,949	1,079	99.85	215.48
Crayfish22	7.44	56.87	24.86	2.72	1.57	67,470	1,438	99.76	239.48
Crayfish23	7.37	58.37	18.51	2.36	1.08	71,574	1,004	99.77	173.11

### Sediment Fungal α-Diversity and the Taxonomic Distribution in Ponds

**Figure 2:**
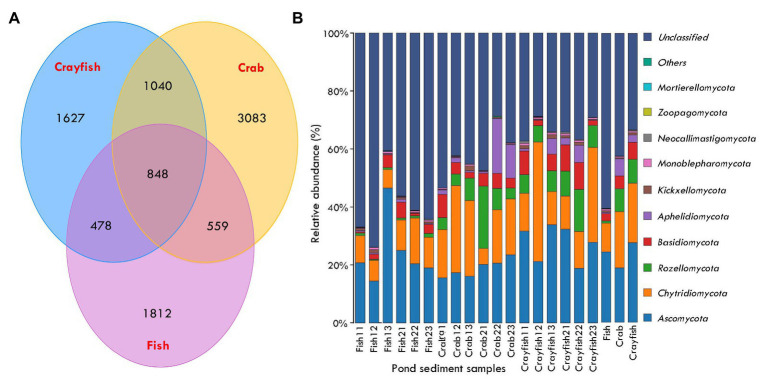
Taxonomic distributions and compositions of sediment fungal communities in aquaculture ponds. **(A)** Venn diagram generated from ITS gene sequencing data illustrating sediment fungal communities that are shared between different aquaculture ponds; **(B)** sediment fungal community compositions at the phylum levels in aquaculture ponds. The last three bars represent averaged sediment fungal community compositions for each type of pond.

In total, 1,290,605 raw sequences were obtained from sediment samples in this study and 1,283,490 remained after quality control and sequence filtering. Sequence number of sediment samples ranged between 66,297 and 74,085 (mean, 71,305 ± 2,356; Table 1). Phylotype richness equivalent to the number of OTUs ranged between 892 and 1,663, and phylogenetic diversity ranged between 171.13 and 450.09 in the sediments (Table 1). The coverage, an index of the captured diversity, ranged between 99.67 and 99.85%, revealing enough sequencing depth of sediment samples to meet the study purposes (Table 1). Lastly, 9,949 OTUs were affiliated into the fungi kingdom across the sediment samples. A total of 848 OTUs were shared by fish, crab, and crayfish ponds (Figure 2A). Crab and crayfish ponds (1,040) had almost twice the shared OTUs compared to fish and crab ponds (478), and fish and crayfish ponds (557), meanwhile the number of unique OTUs was larger in crab ponds (3,083) than fish and crayfish ponds (1,627 and 1,812; Figure 2A).

The taxonomically assigned OTUs from the pond sediments belonged to 15 phyla, 41 classes, 91 orders, 195 families, and 273 genera. The OTUs were mainly classified into *Ascomycota*, with the abundance ranging from 14.4 to 46.5% of total fungal sequences (mean, 23.6 ± 8.1%) and followed by *Chytridiomycota* from 5.5 to 41.2% (16.6 ± 9.9%) and *Rozellomycota* from 0.3 to 21.4% (5.7 ± 5.4%; Figure 2B). To a lesser extent, other fungi phyla were *Basidiomycota* (4.4 ± 2.9%) and *Aphelidiomycota* (2.9 ± 4.9%). The unclassified fungi presented the most abundance in pond sediments, ranging between 28.7 and 73.9% (45.5 ± 14.1%; Figure 2B). The *Ascomycota* had a greater abundance of sediments from crayfish ponds compared to crab and crayfish ponds. Conversely, the *Chytridiomycota*, *Rozellomycota*, *Basidiomycota*, and *Aphelidiomycota* presented lower abundance in sediments from fish ponds (Figure 2B). The abundance of sediment unclassified fungi showed significant differences among the ponds and the fish pond sediments had the highest unclassified fungi abundance (60.5 ± 11.4%), followed by crab ponds (42.5 ± 8.5%) and crayfish ponds (33.4 ± 3.6%; Figure 2B).

**Figure 3:**
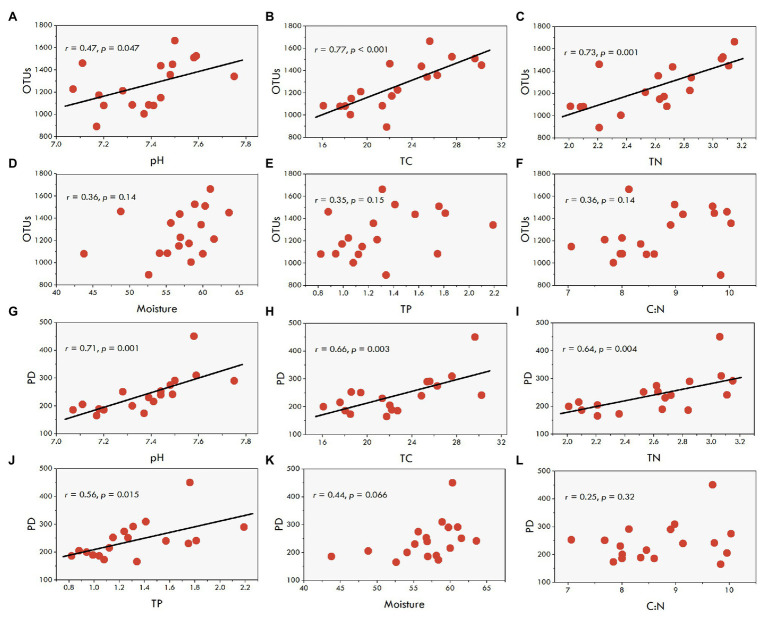
The correlations between sediment fungal diversity and the environmental factors in aquaculture ponds. (**A**-**F**), the correlations of fungal OTU with pH, TC, TN, Moisture, TP, and C:N, respectively. (**G**-**L**), the correlations of fungal PD with pH, TC, TN, TP, Moisture, and C:N, respectively.

The results of the regression analysis showed that phylotype richness of fungal communities was significantly correlated with sediment pH (*r* = 0.47, *p* = 0.047), TC (*r* = 0.77, *p* < 0.001), and TN (*r* = 0.73, *p* = 0.001; Figures 3A–C), yet it was not significantly correlated with sediment moisture, TP, and C:N ratio (Figures 3D–F). Similarly, the phylogenetic diversity of the fungal communities exhibited a significantly positive relationships not only with sediment pH (*r* = 0.71, *p* = 0.001), TC (*r* = 0.66, *p* = 0.003) and TN (*r* = 0.64, *p* = 0.004), but also with TP (*r* = 0.56, *p* = 0.015; Figures 3G–J). The correlations of the phylogenetic diversity with sediment moisture and C:N ratio were not significant (Figures 3K,L).

### Sediment Fungal Community Structure

**Figure 4:**
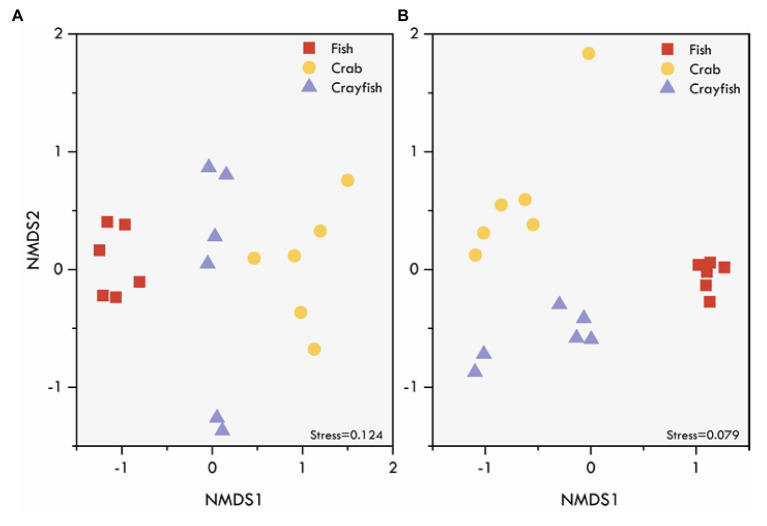
Differences in fungal community structure of aquaculture pond sediments as indicated by NMDS ordination performed on **(A)** Bray-Curtis and **(B)** Jaccard dissimilarity matrices. Different symbols represent different aquaculture pond sediment samples.

NMDS ordinations based on Bray-Curtis distance revealed the differences in fungal community structure among the pond sediments (Figure 4). Markedly, there was a clear distinction among the sediment samples of fish, crab, and crayfish ponds, suggesting the higher similarities of the fungal community structure of sediments collected from the same type of ponds than those in different types of ponds. The NMDS based on Jaccard distance also showed a similar pattern of clustering among the sediment samples. Significant differences of the sediment fungal communities were further supported by two multivariate statistical tests, PERMANOVA and ANOSIM, based on both Bray-Curtis dissimilarity and Jaccard distance which both simultaneously showed that the community differences were statically significant among the ponds (Table 2). Moreover, the crab and crayfish ponds showed a higher sediment fungal community dissimilarity than that between fish and crab, and fish and crayfish ponds (Table 2). These results indicate sediment fungal community assemblages depend on farming practices in aquaculture ponds.

**Table 2:** Fungal community dissimilarity comparisons among different pond sediments using two non-parametric statistical methods.

Distance matrix	Pond sediments	PERMANOVA		ANOSIM	
	Multiple comparisons	*R*^2^	*p*	*R*	*p*
Bray-Curtis dissimilarity	Fish vs. crab vs. crayfish	0.264	0.001	0.728	0.001
	Fish vs. crab	0.245	0.001	0.959	0.002
	Fish vs. crayfish	0.225	0.003	0.652	0.004
	Crab vs. crayfish	0.166	0.001	0.439	0.002
Jaccard dissimilarity	Fish vs. crab vs. crayfish	0.218	0.001	0.851	0.001
	Fish vs. crab	0.190	0.001	0.883	0.002
	Fish vs. crayfish	0.193	0.001	0.961	0.003
	Crab vs. crayfish	0.136	0.003	0.635	0.004

### The Driving Factors for Sediment Fungal Communities

**Figure 5:**
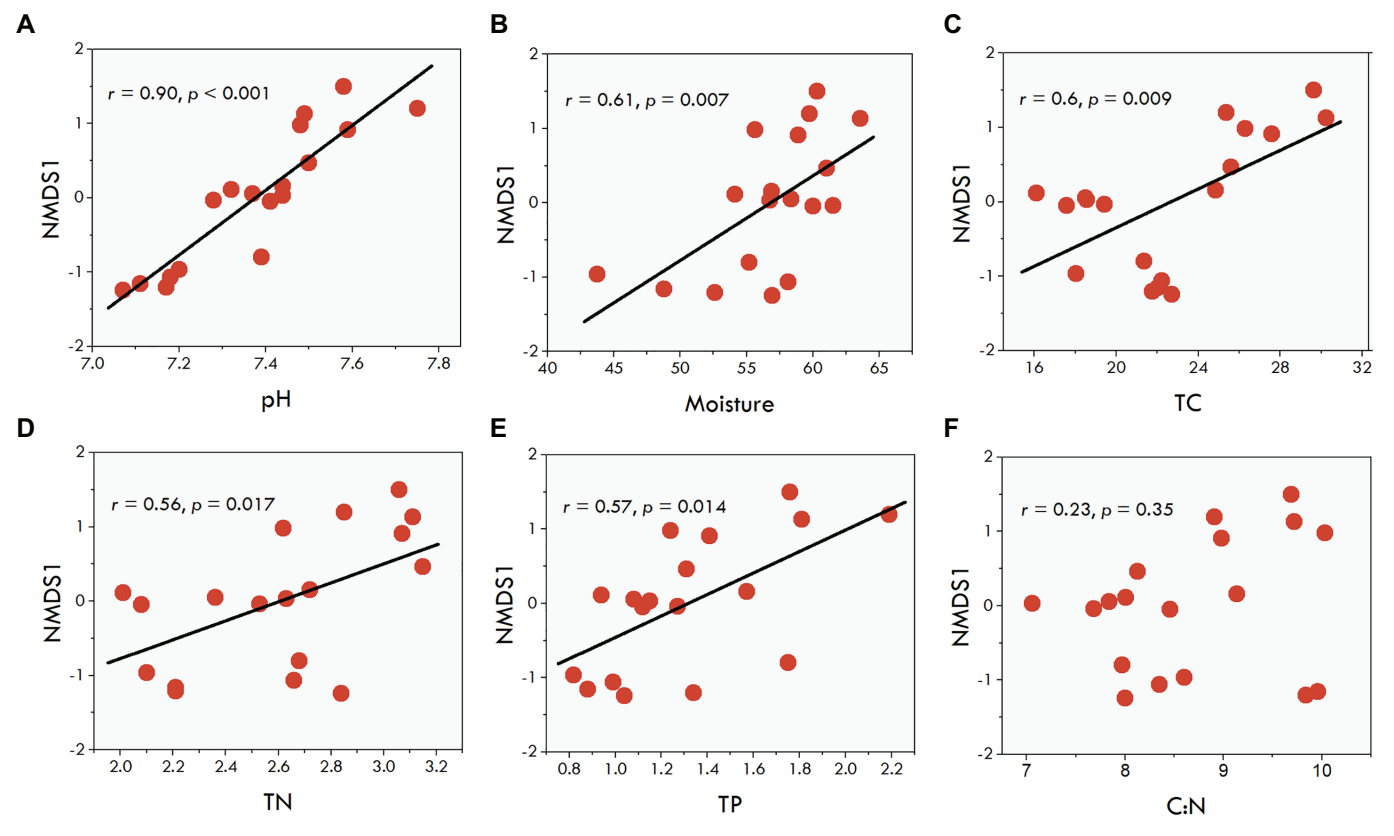
The correlations between the sediment fungal NMDS1 scores (first axis) based on Bray-Curtis distances and environmental factors. (**A**-**F**), the correlations of fungal OTU with pH, TC, TN, Moisture, TP, and C:N, respectively.

Further, we explored the potential factors associated with farming practices for driving fungal communities in the sediments. Six sediment variables (sediment pH, moisture, TC, TN, TP, and C:N ratio) were used to explain the variation in the observed fungal community structure. The regression analysis between the NMDS scores and various sediment environmental variables revealed significant relationships of sediment variables with NMDS1 (Figure 5 and Supplementary Table S1), but not with NMDS2 (Supplementary Table S1). Except for the C:N ratio, the results showed sediment pH (*r* = 0.90, *p* < 0.001), moisture (*r* = 0.61, *p* = 0.007), TC (*r* = 0.60, *p* = 0.009), TN (*r* = 0.54, *p* = 0.017), and TP (*r* = 0.57, *p* = 0.014) were positively related to NMDS1 (Figures 5A–E), suggesting that differences in the fungal communities were primarily determined by sediment pH and moisture, and the nutrients along the NMDS 1 axis. Subsequently, as reported above, a subset of sediment properties was used for the correlation analysis with fungal communities. The results showed that the selected sediment variables had a significant correlation with the fungal community structure (Mantel test, *r* = 0.19, *p* = 0.013). The community structure was mainly associated with sediment pH (Mantel test, *r* = 0.23, *p* = 0.004) and TC (Mantel test, *r* = 0.37, *p* = 0.001).

### Prediction of Fungal Ecological Functions

Fungal trophic modes and functional groups of sediment fungal communities were referred using FUNGuild. In this study, 30.83 ± 9.44% of OTUs sequences were assigned into seven trophic modes. Of them, sediment fungi from two trophic modes, saprotroph and pathotroph-symbiotroph, had significantly more sequences in sediments from crayfish ponds (0.64 ± 0.37% and 22.35 ± 4.92%) than those from fish ponds (0.15 ± 0.07% and 14.33 ± 4.24%) and crab ponds (0.29 ± 0.18% and 12.70 ± 1.50%; Table 3). The five other trophic modes had no significant differences among the three types of pond sediments. The fungi that were classified into unidentified trophic modes had a significantly lower proportion in sediments from crayfish ponds (58.45 ± 10.22%), followed by fish ponds (72.85 ± 7.98%) and crab ponds (76.22 ± 2.63%; Table 3). Regarding the fungal function, a total of 14 fungal functional guilds were detected in the seven trophic modes (Supplementary Table S2). Animal endosymbiont-plant saprotroph fungi accounted for the majority (47 ± 22%). The sediments of crab ponds had the lowest abundance of animal endosymbiont-plant saprotroph fungi (22 ± 12%), compared to those in fish ponds (61 ± 14%) and crayfish ponds (58 ± 18%). Plant pathogen-wood saprotroph, undefined saprotroph, and wood saprotroph mainly accounted for the remainder and presented the abundant differences in sediments among the ponds (Supplementary Table S2).

**Table 3:** Relative abundance of fungal sequences classified by the corresponding fungal trophic mode in sediments of different aquaculture ponds in the middle Yangtze River basin, inferred by FUNGuild.

Trophic mode	Fish	Crab	Crayfish	*p*
Saprotroph-symbiotroph	0.11 ± 0.06	0.05 ± 0.03	0.16 ± 0.12	ns
Saprotroph	0.15 ± 0.07^a^	0.29 ± 0.18^a^	0.64 ± 0.37^b^	0.038
Pathotroph-symbiotroph	14.33 ± 4.24^a^	12.7 ± 1.5^a^	22.35 ± 4.92^b^	0.034
Pathotroph-saprotroph-symbiotroph	4.48 ± 7.67	0.47 ± 0.32	0.07 ± 0.05	ns
Pathotroph-saprotroph	4.02 ± 2.63	1.83 ± 1.11	1.59 ± 1.4	ns
Pathotroph	0.38 ± 0.17	0.97 ± 0.34	1.18 ± 0.73	ns
Pathogen-saprotroph-symbiotroph	3.66 ± 1.61	7.39 ± 2.24	15.53 ± 12.22	ns
Unclassified fungi	72.85 ± 7.98^a^	76.22 ± 2.63^a^	58.45 ± 10.22^b^	0.028

Different small letters represent the significant differences; ns, no significant different.

## Discussion

The present study was to investigate sediment fungal communities in aquaculture ponds and explore the fungal community patterns associated with species-based farming practices. This is the first attempt to reveal the previously unknown fungal community of sediments from three types of freshwater aquaculture ponds using a high-throughput sequencing technique, benefiting the identification and differentiation in the fungal communities in the ponds with different farming practices. A high level of diverse OTU composition was observed in the sediments of aquaculture ponds. Fish, crab, and crayfish ponds showed significantly different patterns of sediment fungal communities and similar community structure in the sediments from the same type of farming ponds at different locations, potentially indicating the community patterns associated with cultured species in the ponds and the obtained community differences independent on regional distribution in this study. In addition, we found that sediment nutrients – especially carbon content – are the important factors affecting the diversity and distribution of fungal communities. Sediment pH within small variations is significantly correlated with changes in the fungal community. This further suggests that the sediment fungal community is predominantly affected by farming practices associated with the resulting substrate properties in aquaculture ponds.

It has been recognized that ponds can harbor considerably more species, and specifically more scarce species, than other types of freshwater systems (Céréghino et al., 2008). A handful of pond studies focus on fungal communities and their relationships with the environmental variables (Guo et al., 2015; Brodie et al., 2018). The study by Guo et al. (2015) explored marine fungal communities in pond farming systems, revealing that more than half of fungal OTUs were potentially novel. However, a total of 131 OTUs were observed in their study by the first-generation sequencing method. This is significantly less than that obtained in our study. Recently, despite the use of a high-throughput sequencing platform, the relatively low number of OTUs for fungi were also detected in sediments from other aquatic ecosystems, such as 190 OTUs in sediments from actually established circular polyethylene tanks (Brodie et al., 2018), 420 OTUs from deep-sea (Zhang et al., 2016) and 1,181 OTUs from mangroves areas (Luis et al., 2019). In previous studies, experimental protocols including the primers, sequencing platforms, and sequencing processing were commonly different. These might directly hinder comparative analysis of fungal communities among the aquatic ecosystems; however, they play a central part in expanding our understanding of fungal communities from various environmental samples (Bärlocher and Boddy, 2016). As expected, sediments in aquaculture ponds are an extraordinary habitat harboring high fungal diversity. Our study, therefore, gives a glimpse into the aquatic fungi thriving in the sediments of aquaculture ponds.

We attempted to explore the community patterns in the ponds associated with different cultured animals. There were more shared taxa between crab and crayfish ponds compared to any two other types of ponds, indicating the potential habitats for some fungal growth are associated with shellfish farming. Particularly, the taxa from *Rozellomycota* are consistently accounted for as having significantly higher abundance in crab and crayfish ponds than fish ponds. More fungal species were unique to crab ponds compared to fish and crayfish ponds, in support of some fungi living in specific habitats. In addition, one of the most interesting findings was the discovery of a considerable unclassified fungal population, highlighting the poor available ITS database coverage of the currently available dataset and the highly novel fungal phylotypes that are yet to be deciphered in aquaculture pond ecosystems. The dominance of unclassified fungi was more obvious in the sediments of fish ponds compared to crab and crayfish ponds. The differences are likely to be disturbances dependent and ecologically significant, as the environmental evolution of fungi and the involvement in ecological function were proposed in previous studies (Jebaraj et al., 2012; Manohar et al., 2015).

In this study, we observed more than 10 fungal phyla and the taxonomic composition mainly comprises fungi from *Ascomycota* and *Chytridiomycota*. This is consistent with previous findings that most fungi belong to the two fungal phyla in many aquatic ecosystems and less abundance of *Basidiomycota* is observed (Bärlocher and Boddy, 2016; Grossart and Rojas-Jimenez, 2016). A previous study by Jones et al. (2011) reported that the members of *Cryptomycota*, i.e., *Rozellomycota* recovered from diverse habitats such as marine and freshwater sediments and some oxygen-depleted environments with very low abundance, and even commonly found in aquatic habitats. In this study, *Rozellomycota* accounted for a significantly high fraction of sediment fungal community in aquaculture ponds, but a few fungi from this phylum were isolated. A recent review of fungi in aquatic ecosystems stresses the dominance of *Rozellomycota* in wastewater treatment plants and sludge digesters (Bärlocher and Boddy, 2016). Aquatic fungi from tropical zones can be divided into marine or freshwater and they had little overlap in taxa in brackish water (Hyde and Alias, 2000; Cai et al., 2006; Jones et al., 2009). Fungi play a wide array of ecological roles such as decomposers, pathogens, or parasites of aquatic organisms and even other fungi (Bärlocher and Boddy, 2016; Cunliffe et al., 2017). The communities from different habitats are driven by the substrate they grow on or reside in, such as senescent leaves, sand, and wood in freshwater streams (Wong et al., 1998; Raja et al., 2009).

Aquaculture ponds are man-made, aquatic ecosystems, and they are generally over-nourished by introducing numerous wasted feeds due to farming practices. Our results support the fact that aquaculture induces high nutrient loadings in pond sediments. The sediments are different in many natural substrates (for example, lakes, reservoirs, and wetlands) belonging to oligotrophic status (Xiong et al., 2012; Wu et al., 2013), indicating habitat differences between lentic ponds and lakes. Similarly, in heavily disturbed habitats, fish, crab, and crayfish ponds had significantly different sediment properties, further revealing the effects of species-based farming practices on the pond substrates. Eventually, the sediment nutrients result in variations in fungal communities among the aquaculture ponds. The impacts of environmental variables on the fungal community were well documented. However, it is limited to a series of studies on terrestrial ecosystems (Geml et al., 2014; Liu et al., 2015; Vasco-Palacios et al., 2019; Zeng et al., 2019), and some lotic-like habitats (Wu et al., 2013; Tisthammer et al., 2016). A large pH gradient (from about 4 to 8 units) weakly affected fungal alpha diversity, but not overall community composition in an arable soil; conversely, the diversity, and in some case community in black soils were strongly associated with soil carbon content, followed by the pH (Liu et al., 2015). This indicates different influences of pH on the community distribution patterns (Geml et al., 2014). In contrast to terrestrial soils with wide pH gradients from very acidic to neutral, and to very alkaline (Rousk et al., 2010; Liu et al., 2015; Vasco-Palacios et al., 2019), sediments are slightly acidic to weakly alkaline environment with narrow pH intervals (Xiong et al., 2012; Wu et al., 2013). This can be attributable to high contents of sediment moisture, as detected in this study, and the imperceptible and continuous water exchange by sediment interface. Despite the small pH gradient in this study, we still found significant effects of sediment pH on the fungal richness and phylogenetic diversity in aquaculture ponds. Recently, Tian et al. (2018) found significantly elevated fungal OTUs richness with increasing pH (7.71–8.84) in alkaline sediments of freshwater lakes. Evidence from environmental pH change (2.45–2.89) in acidic soils of three Amazon forests supports the differences in fungal communities (Vasco-Palacios et al., 2019). It seems that these findings are contrasting with results of a wide pH optimum for fungal growth in previous studies (Wheeler et al., 1991; Nevarez et al., 2009). However, it must be recognized that only a few fungal species or some specific taxa were used for pure cultures and therefore they might not completely represent other fungal growth trajectories and the real status of the fungal community in nature.

In spite of the striking effects of pH on fungal communities, the mechanisms for the fungal responses can be expected: a hypothesis is that environmental pH triggers fungal physiological changes inducing alterations of fungal growth and competitive outcomes of fungal taxa. In addition, sediment pH might cooperate with the nutrients, salinity, and other environmental factors such as various metal cations due to the potential relationships (Brady and Weil, 2007). In this study, sediment nutritional factors explained some differences in sediment fungal communities of ponds at a small scale and carbon content was the dominant determining factor shaping the fungal community. In parallel with the study by Tian et al. (2018), the authors provided evidence that sediment carbon was one of the best predictors for driving fungal communities in small lakes distributed in the Headwater Region of Yellow River. It means that the characteristics of sediment nutrients driven by species-based farming practices can affect fungal community assembly (Guo et al., 2015) and that carbon content may robustly shape fungal communities against other nutritional variables. In addition, nitrogen, phosphorus, and other variables regulate widespread, locally abundant fungi in sediment from various ecosystems such as lakes and rivers over broad spatial scales (Xiong et al., 2012; Wu et al., 2013; Tian et al., 2018). Integrating the findings, this study consequently indicates the common effects of sediment environmental factors on fungi across biomes.

## Conclusion

The present study represents an attempt to investigate the fungal community in sediments across different aquaculture ponds. We found that the pond sediments appear to harbor a high fungal diversity and an unusual fungal community with numerous unidentified fungi and the dominant *Ascomycota* and *Chytridiomycota*. The distribution patterns of the fungal communities are obviously dependent on species-based farming practices in the ponds. The community structure and diversity are largely driven by sediment pH and nutrients especially sediment carbon content. This study provides an essential set of information on sediment fungal communities in aquaculture ponds. Although the study is limited to small local scales, it highlights the poorly understood and emerging artificial pond habitats for fungi thriving in the ecosystems. From a practical point and large-scale perspectives, future work in this field should consider sediment fungi within pond context under different farming practices alongside understanding the driving factors and functional roles within individual ecosystems and predicting large-scale fungal responses to aquaculture activities.

## Data Availability Statement

Publicly available datasets were analyzed in this study. This data can be found at: PRJNA611895.

## Author Contributions

ZZ, CS, and YZ designed the experiment. ZZ and QD conducted the experiment and analyzed the data. ZZ and CS wrote the manuscript. XC revised the manuscript. All authors contributed to the article and approved the submitted version.

### Conflict of Interest

The authors declare that the research was conducted in the absence of any commercial or financial relationships that could be construed as a potential conflict of interest.
