# Supplementary material for: Patterns of Sediment Fungal Community Dependent on Farming Practices in Aquaculture Ponds
Source: Front Microbiol. 2021 Feb 19;12:542064. doi: 10.3389/fmicb.2021.542064 (PMC7933557; doi:10.3389/fmicb.2021.542064)
Supplement: Supplementary file 1 [file Data_Sheet_1.pdf]

# Supplemental materials

## Patterns of sediment fungal community dependent on farming practices in aquaculture ponds

**Zhimin Zhang[a], Qinghui Deng[a,b], Xiuyun Cao[a], Yiyong Zhou[a], Chunlei Song[a,]***

[a] *Key Laboratory of Algal Biology, State key laboratory of Freshwater Ecology and Biotechnology, Institute of Hydrobiology, Chinese Academy of Sciences, Wuhan 430072, P. R. China*

[b] *University of Chinese Academy of Sciences, Beijing 100039, P. R. China*

*Corresponding author: Dr. Chunlei Song; E-mail address: clsong@ihb.ac.cn

**Table 1S**. The pairwise regression analyses between the NMDS scores and various sediment parameters. Values in bold indicate significant correlations ($P < 0.05$).

| Sediment parameters | NMDS (Bray-Cirtus) | | | |
|---|---|---|---|---|
| | NMDS1 | | NMDS2 | |
| | r | p | r | p |
| pH | 0.9 | < **0.001** | 0.07 | 0.78 |
| Moisture | 0.61 | **0.007** | -0.026 | 0.92 |
| TC | 0.6 | **0.009** | 0.24 | 0.34 |
| TN | 0.56 | **0.017** | 0.27 | 0.28 |
| TP | 0.57 | **0.014** | 0.22 | 0.37 |
| C:N | 0.23 | 0.35 | 0.097 | 0.7 |

**Table 2S.** The relative abundance (%, sequences) of assigned fungal functional guild in different trophic modes for different types of pond sediments around Hong Lake, in the middle Yangtze River Basin, China, inferred by FUNGuild.

| Trophic mode | Guild | Fish | Crab | Crayfish |
|---|---|---|---|---|
| Saprotroph-Symbiotroph | Animal Endosymbiont-Plant Saprotroph | 0.61±0.14 | 0.22±0.12 | 0.58±0.18 |
| Symbiotroph | Arbuscular Mycorrhizal | 0.02±0.02 | 0.05±0.04 | 0.01±0.01 |
| Saprotroph | Dung Saprotroph | 0±0 | 0.08±0.05 | 0±0 |
| Pathotroph-Saprotroph-Symbiotroph | Dung Saprotroph-Endophyte-Wood Saprotroph | 0±0 | 0.13±0.09 | 0±0 |
| Saprotroph | Dung Saprotroph-Undefined Saprotroph | 0±0 | 0±0 | 0±0 |
| Symbiotroph | Ectomycorrhizal | 0.04±0.02 | 0.03±0.02 | 0±0 |
| Symbiotroph | Endophyte | 0.01±0.01 | 0.04±0.04 | 0±0 |
| Symbiotroph | Lichenized | 0.04±0.03 | 0.12±0.09 | 0.17±0.17 |
| Symbiotroph | Orchid Mycorrhizal | 0±0 | 0.01±0.01 | 0±0 |
| Pathogen | Plant Pathogen | 0±0 | 0±0 | 0.16±0.16 |
| Pathotroph-Saprotroph | Plant Pathogen-Wood Saprotroph | 0±0 | 0.04±0.03 | 0.03±0.03 |
| Saprotroph | Undefined Saprotroph | 0.18±0.16 | 0.19±0.16 | 0.05±0.04 |
| Saprotroph | Undefined Saprotroph-Wood Saprotroph | 0.08±0.05 | 0.01±0.01 | 0±0 |
| Saprotroph | Wood Saprotroph | 0.02±0.02 | 0.09±0.04 | 0.01±0.01 |
